# ACAULIS5 Is Required for Cytokinin Accumulation and Function During Secondary Growth of *Populus* Trees

**DOI:** 10.3389/fpls.2020.601858

**Published:** 2020-11-16

**Authors:** Ana Milhinhos, Benjamin Bollhöner, Miguel A. Blazquez, Ondřej Novák, Célia M. Miguel, Hannele Tuominen

**Affiliations:** ^1^Umeå Plant Science Centre, Department of Plant Physiology, Umeå University, Umeå, Sweden; ^2^Instituto de Biología Molecular y Celular de Plantas, Consejo Superior de Investigaciones Científicas – Universidad Politécnica de Valencia, Valencia, Spain; ^3^Umeå Plant Science Centre, Department of Forest Genetics and Plant Physiology, Swedish University of Agricultural Sciences, Umeå, Sweden; ^4^Laboratory of Growth Regulators, Faculty of Science, Institute of Experimental Botany, Czech Academy of Sciences, Palacký University Olomouc, Olomouc, Czechia; ^5^Instituto de Tecnologia Química e Biológica António Xavier, Universidade Nova de Lisboa, Oeiras, Portugal; ^6^Instituto de Biologia Experimental e Tecnológica (iBET), Oeiras, Portugal; ^7^Biosystems & Integrative Sciences Institute (BioISI), Faculdade de Ciências, Universidade de Lisboa, Lisboa, Portugal

**Keywords:** *ACAULIS5*, cytokinin, *POPACAULIS5*, polyamine, *Populus tremula* × *Populus tremuloides*, thermospermine, wood development, xylem

## Abstract

In the primary root and young hypocotyl of *Arabidopsis*, ACAULIS5 promotes translation of SUPPRESSOR OF ACAULIS51 (SAC51) and thereby inhibits cytokinin biosynthesis and vascular cell division. In this study, the relationships between ACAULIS5, SAC51 and cytokinin biosynthesis were investigated during secondary growth of *Populus* stems. Overexpression of *ACAULIS5* from the constitutive *35S* promoter in hybrid aspen (*Populus tremula* × *Populus tremuloides*) trees suppressed the expression level of *ACAULIS5*, which resulted in low levels of the physiologically active cytokinin bases as well as their direct riboside precursors in the transgenic lines. Low *ACAULIS5* expression and low cytokinin levels of the transgenic trees coincided with low cambial activity of the stem. ACAULIS5 therefore, contrary to its function in young seedlings in *Arabidopsis*, stimulates cytokinin accumulation and cambial activity during secondary growth of the stem. This function is not derived from maturing secondary xylem tissues as transgenic suppression of *ACAULIS5* levels in these tissues did not influence secondary growth. Interestingly, evidence was obtained for increased activity of the anticlinal division of the cambial initials under conditions of low *ACAULIS5* expression and low cytokinin accumulation. We propose that ACAULIS5 integrates auxin and cytokinin signaling to promote extensive secondary growth of tree stems.

## Introduction

The activity of the vascular cambium is coordinated by the action of several different plant hormones during secondary growth of plants. Auxin and cytokinins interact in maintaining the cambial cell identity and cell division activity (for a recent review see Fischer et al., 2019). The endogenous auxin indole-3-acetic acid (IAA) shows highest concentration in the xylem side of the vascular cambium (Tuominen et al., 1997) while cytokinins peak in the phloem (Immanen et al., 2016). Also, gibberellins, ethylene and strigolactones are involved in various aspects of cambial growth (Agustí et al., 2011; Ragni et al., 2011; Etchells et al., 2012). The work with the *Arabidopsis thaliana acl5* (*ACAULIS5*) mutant, which hosts a mutation in a thermospermine synthase, has established a role also for thermospermine in xylem development. The *acl5* mutant has thick leaf veins, enhanced proliferation of xylem cells in young hypocotyls and inflorescence stems and increased stele size of the primary roots suggesting a role for ACL5 or thermospermine in suppressing cell division activity of *Arabidopsis* leaves, inflorescence stems, hypocotyls and primary roots (Hanzawa et al., 1997, 2000; Clay and Nelson, 2005; Knott et al., 2007; Muñiz et al., 2008).

The discovery of suppressor mutants of the *acl5* identified the basic-helix-loop-helix (bHLH) transcription factor SAC51 (SUPPRESSOR OF ACAULIS51) as a downstream target of ACL5. A premature stop codon in an upstream open reading frame (uORF) of SAC51 led to suppression of *acl5* phenotype due to enhanced transcription and translation of the SAC51 main ORF in the *acl5* background (Imai et al., 2006). Several other mutations were later identified in the uORF of SAC51 and the homologous SAC51-like 1 (SACL1) and SACL3 (collectively the “SACL”) as suppressors of *acl5* (Vera-Sirera et al., 2015). Three additional suppressors of *acl5* with mutations in the ribosomal proteins L10, RPL4 and RACK1 suggested involvement of thermospermine in translational regulation (Imai et al., 2008; Kakehi et al., 2015). Collectively, the *acl5* suppressors suggest that ACL5 functions to repress the inhibition of SAC51 translation imposed by its uORF, allowing SAC51 function on its downstream targets, such as genes controlling cell division (Vera-Sirera et al., 2010).

Mechanistic understanding for the ACL5-mediated control of cell division was recently obtained through demonstration of the interaction between SAC51 and the SAC51-like proteins with LONESOME HIGHWAY (LHW). LHW is a bHLH-type transcription factor which interacts with another bHLH transcription factor TARGET OF MONOPTEROS5 (TMO5) to stimulate periclinal cell divisions in the vasculature by activating expression of the cytokinin biosynthetic *LONELY GUY3* (*LOG3*) and *LOG4* genes (de Rybel et al., 2014; Ohashi-Ito et al., 2014). The SACL proteins inhibit interaction of LHW with TMO5 by binding to LHW and hence prevent the action of the LHW-TMO5 dimer to activate the *LOG* genes (Vera-Sirera et al., 2015). Since ACL5 promotes accumulation of the SACL proteins, ACL5 is expected to suppress periclinal cell division by inhibiting cytokinin biosynthesis. Indeed, *acl5* mutants showed increased activity of LHW and increased biosynthesis of cytokinins in the vascular tissues of *Arabidopsis* primary roots and young hypocotyls (de Rybel et al., 2014; Ohashi-Ito et al., 2014; Vera-Sirera et al., 2015). However, this regulatory pathway does not seem to operate during secondary growth of tree stems when ACL5 is required for normal vascular cell division activity rather than suppressing it (Muñiz et al., 2008; Milhinhos et al., 2013). We showed earlier that overexpression of the *Populus trichocarpa ACAULIS5* (*POPACAULIS5*) results in suppression of the *ACAULIS5* expression most probably through co-suppression with concomitant reduction in thermospermine levels. The low *POPACAULIS5* expression level of the transgenic trees resulted in reduced cambial cell division activity of the stem. In the present study, we aimed to clarify the relationship between ACAULIS5, SAC51 and cytokinin biosynthesis during secondary growth of *Populus* stems. Overexpression of *POPACAULIS5* in hybrid aspen (*Populus tremula* × *Populus tremuloides*) trees resulted in suppressed *ACAULIS5* expression (derived from both the *POPACAULIS5* transgene and the endogenous *Populus tremula* × *tremuloides ACAULIS5, PttACL5*). Suppressed *ACAULIS5* expression resulted in low cytokinin levels of the stem and coincided with reduced secondary growth of the stems. ACAULIS5 is therefore required for adequate cytokinin accumulation during secondary growth of the stem.

## Materials and Methods

### Plant Material, Growth Conditions, and Sampling

Hybrid aspen (*Populus tremula* L × *Populus tremuloides* MICHX; clone T89) was maintained by subculture on MS basal salt medium at half strength (Murashige and Skoog, 1962; Milhinhos et al., 2013). Plants were grown in growth chambers at 21°C and 16 h light/8 h dark photoperiod. Transgenic and wild type (WT) T89 plants were transferred to soil and trees were grown for 2 months in the greenhouse at 21°C temperature and 18 h light/6 h dark photoperiod.

For the gene expression analyses and cytokinin quantification, samples from 2-month-old WT T89 control trees and transgenic lines 35S:*POPACAULIS5*-B2, -B4, -B13, -B14, and -B15 and from pCOMT:*PttACL5*-C3, -C7, -C8, -C13, -C15, and -C16 were immediately frozen after harvesting and stored at −80°C until use. The “leaves” sample consisted of the first fully expanded leaves. Samples from stem tissues were obtained between stem internodes 25 and 35 (from the top). A “cambium/phloem” sample was obtained by peeling off the bark and scraping the frozen cells from inside the bark until the emergence of the phloem fibers. “Expanding xylem” sample was collected by scraping the expanding, non-lignified tissues from the surface of the woody tissues. The “mature xylem” sample was collected after removal of the expanding xylem by scraping the maturing (lignifying) tissues until the location of fiber cell death which became apparent as a shift in the color and texture of the wood. The tissues were ground to powder and portioned for gene expression analyses and hormone quantifications.

### Generation of Gene Constructs

Cloning of the *POPACAULIS5* (Potri.006G222200) cDNA and construction of the transgenic 35S:*POPACAULIS5* lines is described in Milhinhos et al. (2013).

The p*COMT*:*PttACL5* lines were created by cloning the *Populus tremula* × *tremuloides PttACL5* (Potrx049518g14918) from T89 cDNA with primers “PttACL5fw gw PU” and “PttACL5rev gw PU,” and recombination by the gateway BP reaction into pDONR207 and, after sequence confirmation, by LR reaction (Karimi et al., 2002) into pK-COMT1P-GW7 (described earlier in Bollhöner et al., 2018). This resulted in a destination vector which allowed expression of *PttACL5* from the xylem specific promoter of the *Populus trichocarpa* CAFFEIC ACID O-METHYLTRANSFERASE (PtCOMT; Tiimonen et al., 2007).

To construct the TCSn:GUS/GFP cytokinin reporter, *TCSn* was amplified from TCSn:min35S:Ω-GFP-ER:nos:302 (Zürcher et al., 2013; a gift from Bruno Müller, University of Zurich, Switzerland) with primers *TCSn_F* and *TCSn_R*. The *TCSn* fragment was first cloned into pCR2.1 and then recombined by BP reaction into pDONR201 with primers *attBs-TCSn-min35S* forward and reverse followed by recombination by LR reaction to pHGWFS7 to produce the TCSn:GUS/GFP construct.

### Hybrid Aspen Genetic Transformation

Expression vectors were transformed into *Agrobacterium tumefaciens* GV3101pMP90 or GV3101pMP90RK strains (Koncz and Schell, 1986) which were used for transformation of hybrid aspen *Populus tremula* × *tremuloides*, as previously described (Nilsson et al., 1992).

Transformant selection from regenerated shoots and shoot elongation was achieved in MS2 medium with MS basal salts, 20 g l^–1^ sucrose, 0.1 μg ml^–1^ indole-butyric acid (IBA), 0.2 μg ml^–1^ 6-benzyl-aminopurine (BAP), 500 μg ml^–1^ cefotaxime and 80 μg ml^–1^ kanamycin monosulphate. After shoot elongation, plants were transferred to half-strength MS medium for rooting.

Transgenic POPACAULIS5 lines B2 and B4, carrying the *35S*:*POPACAULIS5* construct, were transformed with *Agrobacterium* bacteria harboring the TCS*n*:GUS/GFP cytokinin sensor. Ten *Populus* double transformant lines carrying both the 35S:*POPCAULIS5* and the TCS*n*:GUS/GFP constructs were selected in 20 μg ml^–1^ hygromycin and 80 μg ml^–1^ kanamycin monosulphate.

### Histochemical GUS Staining

For histochemical GUS staining, small pieces of stem (1–3 mm × 1–3 mm) were stained in 1 mM X-gluc (5-bromo-4-chloro-3-indol glucuronide) solution containing 1 mM K_3_Fe(CN)_6_, 1 mM K_4_Fe(CN)_6_, 0.1% Triton X-100 in 50 mM NaPO_4_ buffer for 1.5 h. Samples were dehydrated in an increasing ethanol series, fixed in FAA (5% formaldehyde and 5% acetic acid in 50% ethanol), washed with 50% ethanol, and stored temporarily in 70% ethanol. Samples were then embedded in LR White by incubating in 95% ethanol for 15 min, 50% ethanol/50% LR White with 10% PEG400 for 15 min, 100% LR White with 10% PEG400 for 20 min twice, and finally in 100% LR White 10% PEG400 with the accelerator (2–3 drops accelerator to 10 ml 100% LR White 10% PEG400). After polymerization, sections were taken at 14 μm with a microtome. Sections were incubated on slides overnight at 60°C and embedded in Entellan. The sections were imaged with a Zeiss Axioplan II microscope equipped with an AxioCam CCD camera (Zeiss, Jena, Germany).

### Anatomical and Ultrastructural Analysis

Tree height, internode length (an average of ten internodes above the 35th internode), leaf dimensions (an average of leaves 31–35 counting from the first expanded leaf at the apex) and stem diameter at internode 35 and stem base (15 cm above soil) were measured after 2 months of growth in the greenhouse. Stem pieces from the 35th internode were used for anatomical analysis. Consecutive, 15-μm hand-microtome sections were stained with phloroglucinol and toluidine blue-O as in Milhinhos et al. (2013). Sections were observed and images captured as described above. Tree growth, growth parameters and microscopy analysis were performed in at least six independent biological replicates from each genotype and line.

### Electron Microscopy

Samples for electron microscopy analyses were taken from stem segments from the 35th internode, fixed in 2.5% glutaraldehyde in 0.2 M sodium cacodylate buffer, embedded in Spurr resin (Sigma) according to Rensing (2002), and examined with a Hitachi H-7000 transmission electron microscope (TEM, Hitachi, Tokyo, Japan).

### Quantitative Real-Time RT-qPCR

Frozen samples were homogenized in a mortar and total RNA was extracted from 100 mg powder according to Chang et al. (1993). cDNA synthesis was performed on 1 μg of DNase-treated (Ambion TURBO DNA-free) total RNA using Transcriptor HF cDNA synthesis kit (Roche) with oligo-dT primers. RT-qPCR was performed in LightCycler 480 PCR system with LightCycler 480 SYBR Green I Master Mix (Roche Applied Science). Specific primer pairs were designed to generate amplicons of *POPACAULIS5* transgene (Potri.006G222200, Milhinhos et al., 2013), *PttACL5* transgene (Potrx049518g14918), *PttACL5* endogenous gene (Potrx049518g14918), *PttSAC51* (Potrx001612g01242, which corresponds to Potri.007G112800; detailed information on *Populus* SAC51 homolog can be found in Supplementary Figures S1A–C), *PttRR7* (Potrx009276g07408, which corresponds to Potri.016G038000; Nieminen et al., 2008; Ramírez-Carvajal et al., 2008; Immanen et al., 2013), *PttRR6* (Potrx009535g07775, which corresponds to Potri.006G041100; Ramírez-Carvajal et al., 2008; Immanen et al., 2013), *PttLOG7a* (Potrx056249g18289, which corresponds to Potri.005G248900; Immanen et al., 2013) used in detection. The primers for the *POPACAULIS5* and *PttACL5* transgenes detected both the transgene and the endogenous *PttACL5. PttACL5_END* forward primer designed in the 5′UTR, and not present in the construct, enabled detection of the endogenous *PttACL5* gene only. The primers are listed in Supplementary Table S2. The amount of target transcripts was normalized by the ΔΔC_*T*_ method (Livak and Schmittgen, 2001) using wild type as the calibrator samples in each tissue and experimental condition and relative to *Histone H3.3* (Potri.005G072300) reference gene (Xu et al., 2011). For all experiments, the mean of triplicate qPCR reactions was determined and at least three biological replicates were used. The experiments were repeated at least twice.

### Quantification of Cytokinin Levels

Cytokinins (CK) were extracted and isolated from 50 mg of frozen tissues as previously described (Svačinová et al., 2012), including modifications described by Antoniadi et al. (2015). To each extract, the stable isotope-labeled CK internal standards (0,1 pmol of CK bases, ribosides, *N*-glucosides, 0.25 pmol of *O*-glucosides, and 0.5 pmol of nucleotides) were added as a reference. Purified samples were analyzed by a liquid chromatography-tandem mass spectrometry (LC-MS/MS) system consisting of an ACQUITY UPLC I-Class System (Waters) and a Xevo TQ-S (Waters) triple quadrupole mass spectrometer. Quantification was obtained using a multiple reaction monitoring (MRM) mode of selected precursor ions and the appropriate product ion. Three independent biological replicates and three technical replicates were analyzed for each sample.

### Statistical Analysis

Student’s *t*-test (one tailed, assuming unequal variance) was employed to assess significant differences in gene expression, CK contents and tree growth parameters. A significance level of *p* = 0.05 was considered. Statistics were performed using R software package.

## Results

### *POPACAULIS5* Expression From *35S* Promoter Interferes With Tree Growth and Wood Anatomy

**FIGURE 1:**
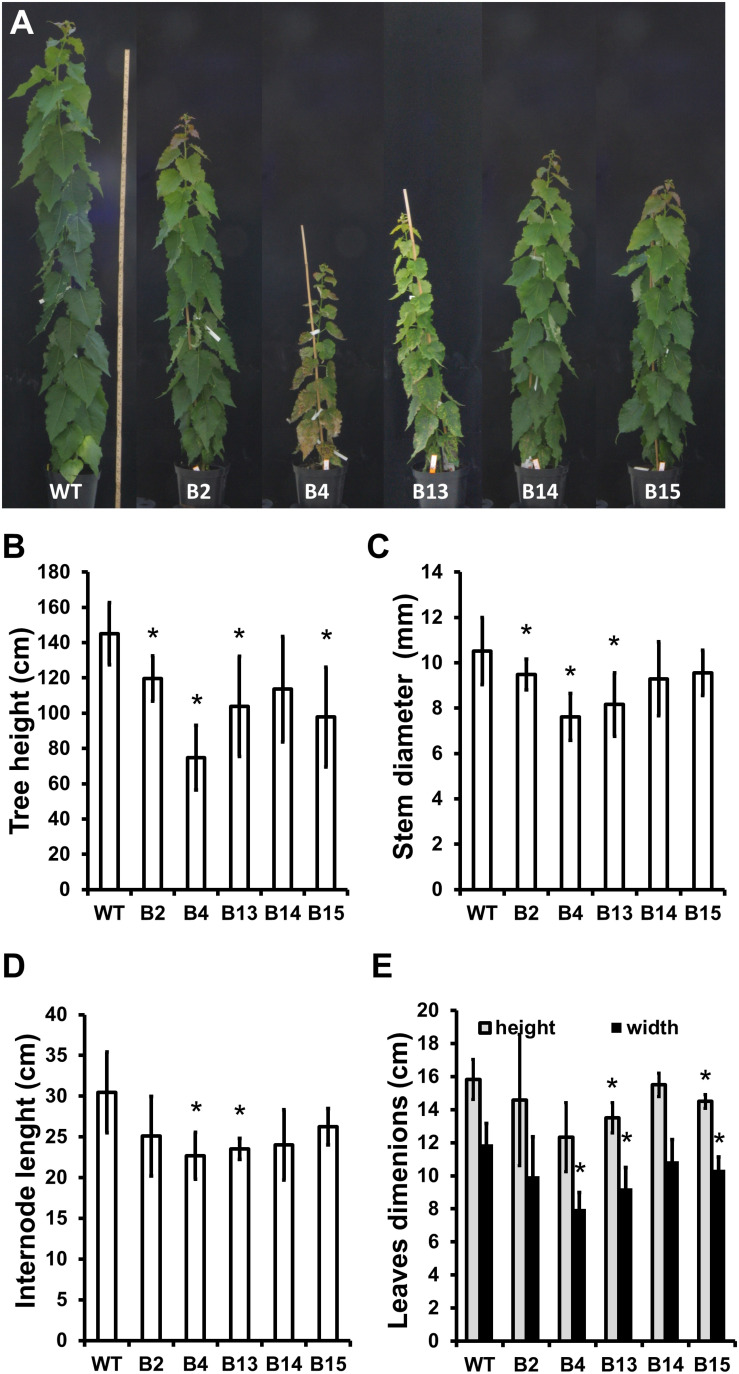
Phenotypic characterization of transgenic hybrid aspen trees expressing *35S*:*POPACAULIS5* construct (“POPACAULIS5” trees). Overall growth **(A)**, tree height **(B)**, stem diameter **(C)**, internode length **(D)** and leaf dimensions **(E)** is shown in 2-month-old wild type (WT) and transgenic POPACAULIS5 trees. Values are means ± SD of at least six biological replicates. The asterisk indicates significant differences from the wild type (*p* < 0.05, Student’s *t*-test).

**FIGURE 2:**
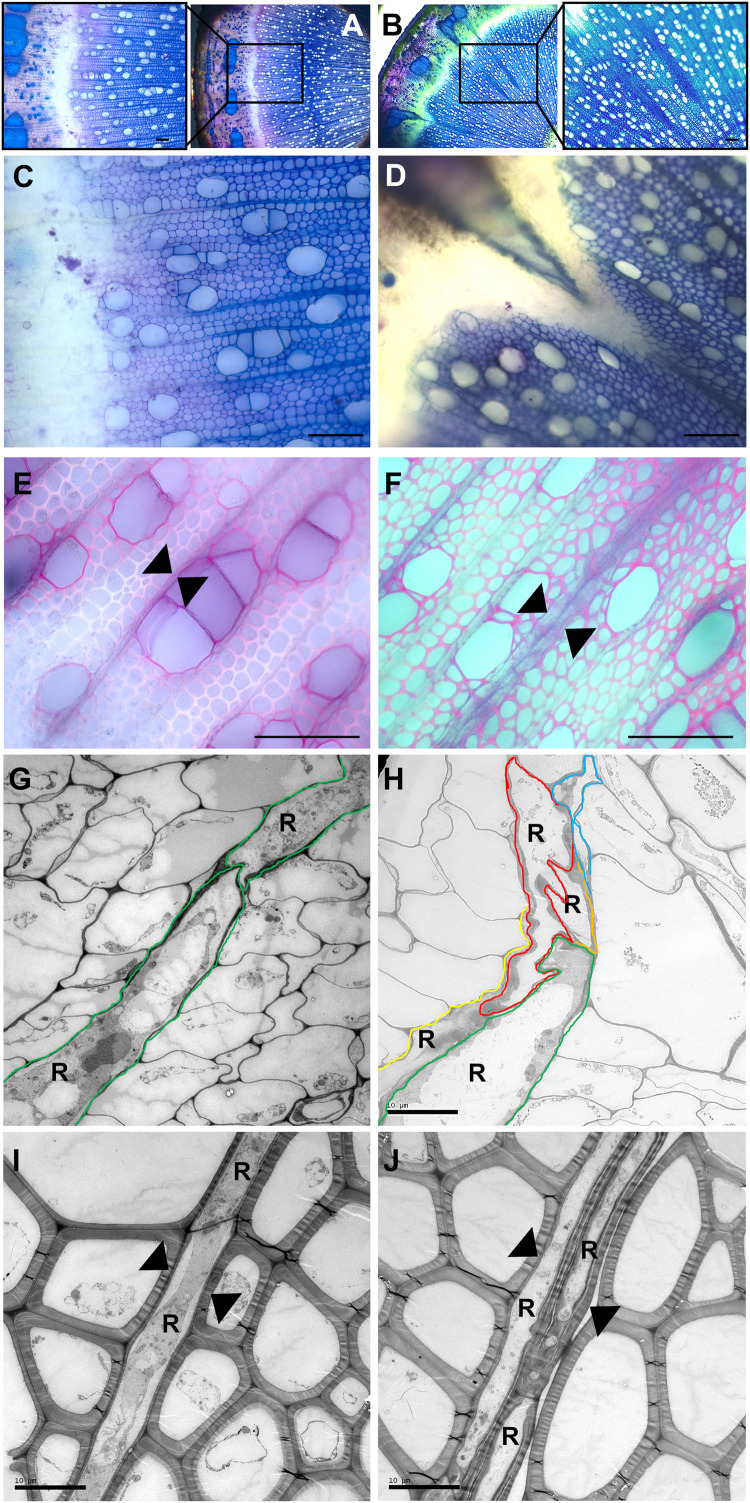
Stem anatomy in wild type and transgenic POPACAULIS5 trees. Transverse sections were taken from the 35th internode from stems of wild type **(A,C,E,G,I)** and transgenic POPACAULIS5 lines B2 and B13 **(B,D,F,H,J)** stained with toluidine blue-O **(A–D)** and phloroglucinol **(E,F)**. Insets in **(A,B)** show increased magnification of stem tissues where disturbances were observed in secondary phloem, cambial zone and secondary xylem in both transgenic POPACAULIS5 lines but not in the WT trees. **(G–J)** Electron micrographs from wild type **(G,I)** and POPACAULIS5 stems **(H,J)**. Colored lines were painted in **(G,H)** to highlight individual cambial ray initial derivatives (R) in the cambial zone. Arrowheads in wild type indicate uniseriate rays whereas biseriate rays were present in the secondary xylem of the transgenic POPACAULIS5 trees. Scale bars: **(A–F)** 100 μm, **(G,H)** 10 μm.

Five transgenic “POPACAULIS5” lines carrying a *35S:POPACAULIS5* construct (Milhinhos et al., 2013) were grown together with the T89 wild type hybrid aspen (*Populus tremula* × *P. tremuloides*) trees in the greenhouse to analyze the function of ACAULIS5 in tree growth. Similar to our earlier report (Milhinhos et al., 2013), both height and diameter growth was suppressed in most of the lines. Line B2 showed least changes in plant height and diameter while lines B4 and B13 were most affected (Figures 1A–C). Also leaf size and internode lengths were affected in the different lines similar to the height and the diameter (Figures 1D,E). In addition, we observed biseriate rays in the secondary xylem tissues of all POPACAULIS5 lines (Figures 2A–J and Supplementary Figures S2A–E). *Populus* wood has normally strictly uniseriate rays (Figures 2A,C,E,G,I). Biseriate rays represented 8.3% (line B14) to 14.4% (line B2) of the total amount of rays found in stem sections of the transgenic lines, whereas they were never detected in wild type controls (Figures 2B,D,F,H,J and (Supplementary Figures S2D,E). A close inspection of xylem anatomy revealed biseriate rays that seemed to originate in the cambium (Figure 2H). The biseriate rays were initiated at different ages of the cambium as both short biseriate rays (Figure 2B) as well as biseriate rays traversing through the whole secondary and secondary phloem were observed (Supplementary Figure S2). In rare cases, the biseriate rays were formed in regions where vascular cambium was discontinuous, resulting in secondary phloem tissues interspersed within the secondary xylem tissues (Figure 2D).

### Modified *POPACAULIS5* Expression Influences Signaling Leading to Cytokinin Biosynthesis

**FIGURE 3:**
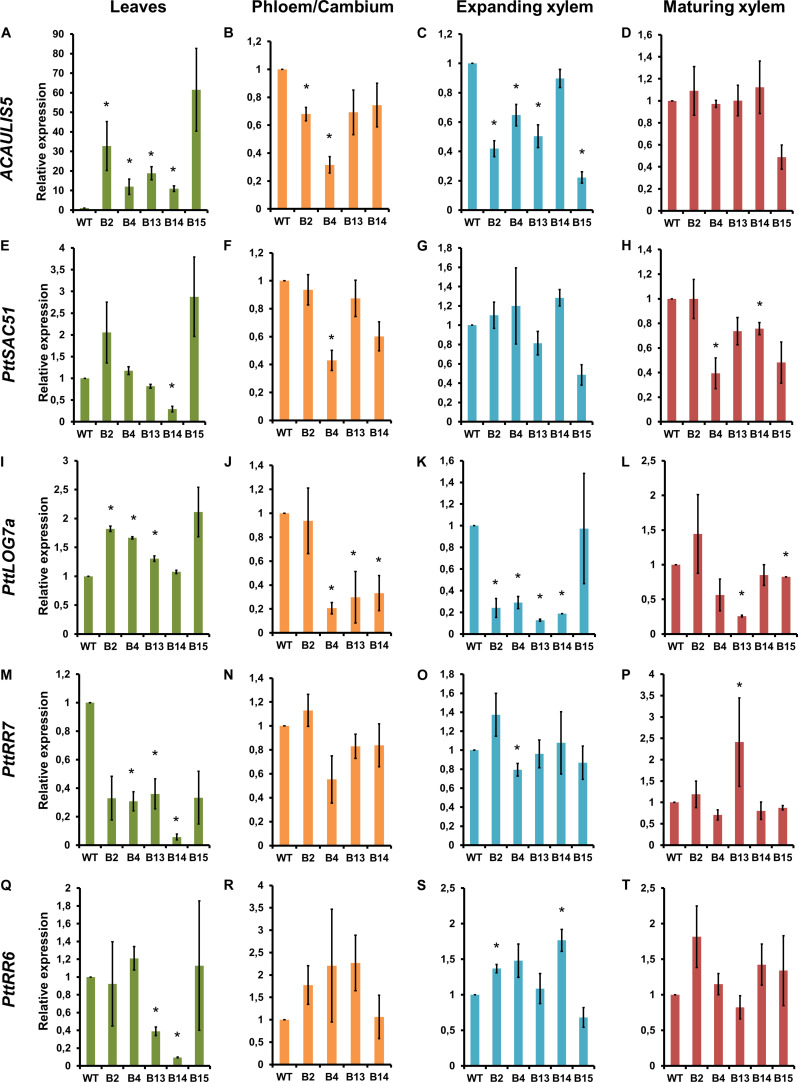
Gene expression analysis in 2-month-old, transgenic POPACAULIS5 trees. Relative gene expression of *ACAULIS5* **(A–D)**, *PttSAC51* **(E–H)**, *PttLOG7a* **(I–L)**, *PttRR7* **(M–P)**, and *PttRR6* **(Q–T)** in leaves, phloem/cambium, expanding xylem and mature xylem tissues of B2, B4, B13, B14. and B15 transgenic POPACAULIS5 lines. Values are means ± SD of three biological replicates and three technical replicates. Transcript levels are given relative to the wild-type level in each tissue. The asterisk indicates significant differences from the wild type (*p* < 0.05, Student’s *t*-test). The experiments for gene expression analyses were repeated twice.

To elucidate the relationship between ACAULIS5, SAC51 and cytokinin biosynthesis, we first analyzed the expression of *ACAULIS5* in four different tissues of the POPACAULIS5 lines using primers that recognized both the *POPACAULIS5* transgene and the endogenous *PttACAULIS5* (*PttACL5*). Increased expression of *ACAULIS5* was observed only in the leaves (Figure 3A). Phloem tissues, which also included the vascular cambium, showed statistically significant reduction of *ACAULIS5* in lines B2 and B4 (Figure 3B). Expanding xylem tissues also showed lower expression of *ACAULIS5* in several lines while maturing xylem tissues did not show statistically significant differences (Figures 3C,D). Decreased expression of *ACAULIS5* is most probably due to co-suppression events, and the expression analysis using primers that detected only the endogenous *PttACL5* demonstrated that also the expression of the endogenous *PttACL5* was suppressed in the different tissues (Supplementary Figures S3A–D). Co-suppression is a phenomenon which seems to be particularly common in the xylem tissues (Endo et al., 2018). The level of co-suppression may vary between cells within a tissue, but the consistency in suppression of *ACAULIS5* expression and concomitant growth phenotypes in experiments extending over several years’ time (see Milhinhos et al., 2013) are indicative of stable co-suppression levels in the different tissues of the transgenic POPACAULIS5 trees. Changes in *ACAULIS5* expression is expected to result in comparable changes in the ACAULIS5 protein as well as thermospermine levels. This is supported by our earlier results showing that POPACAULIS5 leaves with high *ACAULIS5* expression had increased thermospermine levels while POPACAULIS5 woody tissues with low *ACAULIS5* expression had decreased thermospermine levels (Milhinhos et al., 2013).

In *Arabidopsis*, ACAULIS5 stimulates translation of SAC51 but indirect effects on transcription are possible due to the reported regulation of SACL gene transcription by TMO5-LHW (Vera-Sirera et al., 2015). In the POPACAULIS5 trees, enhanced expression of *ACAULIS5* in the leaves of the lines B2 and B15 coincided with enhanced expression of a *Populus* SAC51 homolog, *PttSAC51* (Supplementary Figures S1A–C), but this was not statistically significant (Figure 3E). The expression of *PttSAC51* followed the expression pattern of *ACAULIS5* also in the phloem/cambium sample with line B4 which showed the lowest expression for both genes (Figure 3F). No apparent correlation in the expression levels of *ACAULIS5* and *PttSAC51* was observed between the different transgenic lines in the xylem tissues of the stem (Figures 3G,H).

SAC51 suppresses the expression of the cytokinin biosynthetic *LOG* genes through interaction with LHW and TMO5 in young *Arabidopsis* seedlings (Vera-Sirera et al., 2015). Several *PttLOG* genes are expressed in the cambial region of *Populus* stem (Immanen et al., 2016; Sundell et al., 2017). *PttLOG7a* is a homolog of the *Arabidopsis LOG7* (Immanen et al., 2013) which regulates vascular development redundantly with LOG3 and LOG4 (and maybe some other LOGs) downstream of SAC51 in *Arabidopsis* (de Rybel et al., 2014; Vera-Sirera et al., 2015). The *Populus PttLOG7a* showed differential expression in the POPACAULIS5 trees (Figures 3I–L). While overproduction of *ACAULIS5* coincided with increased expression of *PttLOG7a* in most of the lines in the leaf tissues, low *ACAULIS5* expression in the other tissues correlated mainly with lower expression of *PttLOG7a* in comparison to the wild type. These results suggest, contrary to what has been reported for young hypocotyls and roots in *Arabidopsis*, positive effect of ACAULIS5 on cytokinin biosynthesis during secondary growth of the stem.

Expression of two A-type response regulators, *PttRR7* and *PttRR6*, suggested suppression of the expression of these genes with enhanced expression of *ACAULIS5* in the leaves (Figures 3M–T). No conclusive results were obtained on whether cytokinin signaling was altered in the stem tissues, which could be related to complicated feedback regulation known to target the A-type response regulators (Immanen et al., 2013).

### Suppressed Expression of *POPACAULIS5* Leads to Decreased Levels of Active Cytokinin

**FIGURE 4:**
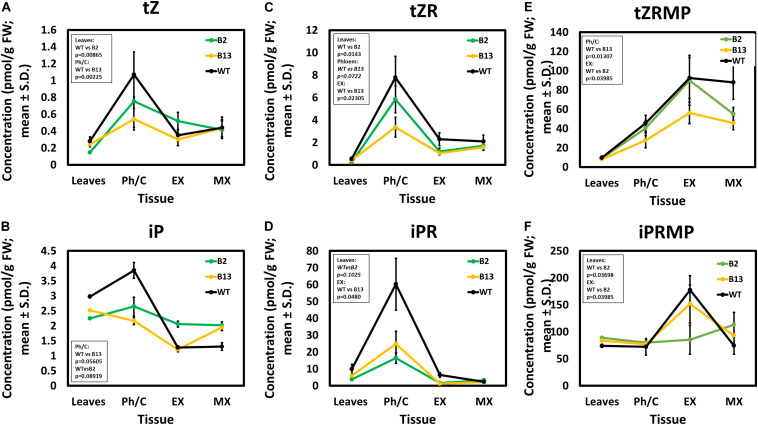
Cytokinin (CK) content in transgenic, 2-month-old POPACAULIS5 trees. Concentration of active cytokinins *trans*-zeatin (*t*Z; **A**), *N*^6^-isopentenyladenine (iP; **B**), their equivalent riboside precursors *trans-*zeatin riboside (*t*Z; **C**) and N^6^-isopentenyladenosine (iPR; **D**), as well as their equivalent nucleotide precursors *trans*-zeatin-riboside 5’-monophosphate (*t*ZRMP; **E**), and N^6^-isopentenyladenosine 5’-monophosphate (iPRMP; **F**), as quantified in leaves and stem tissues of phloem/cambium (Ph/C), expanding xylem (EX) and maturing xylem (MX). Values are means ± SD of three biological replicates and three technical replicates for each biological replicate. Statistically significant differences from wild type when *p* < 0.05 (Student’s *t*-test) are listed in the inset.

Since the *PttLOG7a* expression analysis suggested a regulation of cytokinin levels by ACAULIS5, we next measured the concentrations of the cytokinin bases (*N*^6^-isopentenyladenine, iP, *trans*- and *cis*-zeatin, *t*Z and *c*Z, dihydrozeatin, DZ), their nucleotide and riboside precursors and their catabolic products (*O*- and *N*-glucosides) in two POPACAULIS5 lines and the wild type (Supplementary Table S1). Line B2 was selected due to its mild growth phenotype and line B13 due to its strong growth phenotype. Somewhat surprisingly, the strong overexpression of *ACAULIS5* in the leaves did not influence the levels of the main physiologically active cytokinins iP and *t*Z or the levels of their direct riboside precursors (Figures 4A–F). Only a trend toward a lower level of iP was observed in the leaves of the transgenic lines (Figure 4B). However, the phloem tissues, which included also the vascular cambium, showed statistically significant reductions in the cytokinin levels of both the active bases and the ribosides in both transgenic lines (Figures 4A–D). For *t*Z, iP and *trans*-zeatin riboside (*t*ZR), the reduction was strongest in line B13 (Figures 4A–C). The expanding and maturing xylem tissues displayed only small differences in the levels of the cytokinins. Only minor, mostly non-significant changes were observed for the cytokinin nucleotides *trans*-zeatin riboside-5’-monophosphate (*t*ZRMP) or *N*^6^-isopentenyladenosine-5’-monophosphate (iPRMP) except for statistically significant reductions in the level of *t*ZRMP for line B13 in the phloem/cambium and in the expanding xylem (Figures 4E,F). Taken together, the main physiologically active cytokinins were all present in lower amounts in the main domain of cytokinin accumulation (i.e., phloem/cambium) in the POPACAULIS5 transgenic lines B2 and B13 when compared to the wild type, suggesting that ACAULIS5 is required for appropriate accumulation of cytokinins in these tissues.

Two transgenic lines, lines B2 and B4, were transformed with the cytokinin marker *TCSn*:*GUS/GFP* to analyze whether changes in *ACAULIS5* expression caused alterations in the spatial distribution of cytokinins in stem tissues. Unfortunately, line B13 could not be used since it did not perform well in the tissue culture after genetic transformation. Fourteen transgenic *TCSn*:*GUS/GFP* lines were created in each background (wild type, line B2 and line B4), and 4–6 representative lines were selected after initial screening for detailed analyses. In wild type trees, high GUS activity was present ubiquitously in the young stem tissues of all lines (Supplementary Figure S4A). In mature stem, GUS activity was typically present in the phloem tissues and in the strongest lines also in the cortex (Supplementary Figure S4B). Rays on the phloem side and phloem companion cells had positive signal in all lines. No obvious differences were observed in the spatial distribution of GUS activity in the POPACAULIS5 lines except for that several *TCSn*:*GUS/GFP* lines in the POPACAULIS5 line B4 background seemed to have a lower overall GUS activity compared to the wild type (Supplementary Figure S4B).

### ACAULIS5 Effect on Cambial Activity Is Not Derived From the Secondary Xylem

*ACAULIS5* has functions both in the cambial growth (Figure 1; Muñiz et al., 2008; Vera-Sirera et al., 2015) and in xylem differentiation (Muñiz et al., 2008; Milhinhos et al., 2013). Even though *ACAULIS5* is expressed in the cambial tissues, the main expression domain is in the secondary xylem tissues of aspen trees (Sundell et al., 2017). It was recently reported that xylem vessel elements direct adjacent cells to divide and function as cambial cells (Smetana et al., 2019). We therefore investigated whether the regulation of cambial activity by ACAULIS5 is derived from the secondary xylem tissues. For this purpose, transgenic hybrid aspen trees were created where the expression of the *Populus tremula* × *tremuloides ACAULIS5* (*PttACL5*) was placed under the control of the xylem specific *Populus trichocarpa COMT* promoter (pCOMT; Tiimonen et al., 2007). After initial screening of 14 different lines, six lines carrying the p*COMT:PttACL5* construct were characterized for growth and cytokinin content after 2 months of growth in the greenhouse.

**FIGURE 5:**
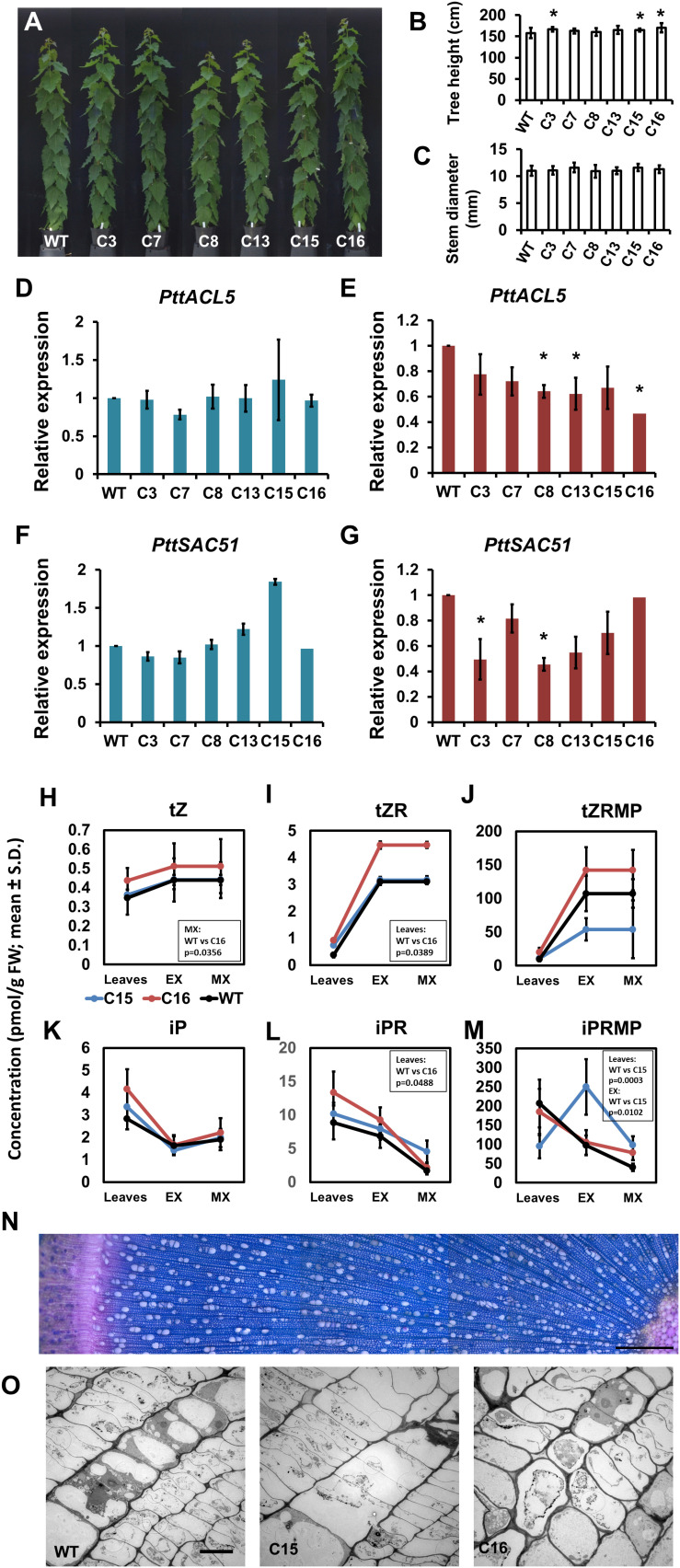
Phenotypic characterization of transgenic trees expressing p*COMT:PttACL5*. Overall growth **(A)**, tree height **(B)** and stem diameter **(C)** is shown for 2-month-old trees of transgenic lines C3, C7, C8, C13, C15, and C16. **(D–G)** Gene expression analysis in secondary xylem tissues of greenhouse grown p*COMT*:*PttACL5* trees. Mean values ± SD of expression is shown for *PttACL5* **(D,E)** and *PttSAC51* **(F,G)** in expanding xylem **(D,F)** and in maturing xylem tissues **(E,G)** relative to the wild type. **(H–M)**. Concentration of cytokinins in wild type (WT) and p*COMT:PttACL5* lines C15 and C16. Mean values for three biological replicates ± SD are shown for *trans*-zeatin (*t*Z; **H**), *trans*-zeatin riboside (*t*ZR; **I**) and *trans*-zeatin riboside-5’-monophosphate (*t*ZRMP; **J**), *N*^6^-isopentenyladenine (iP; **K**), and *N*^6^-isopentenyladenosine (iPR; **L**) and *N*^6^-isopentenyladenosine-5’-monophosphate (iPRMP; **M**) in leaves and expanding secondary xylem (EX) and maturing xylem (MX) of the stem. **(N)** Transverse section of p*COMT*:*PttACL5* line C16 stem evidencing lack of disturbances in wood anatomy, cambial cells or rays. The image is composition of overlapping micrographs taken with 20x objective. Scale bar indicates 500 μm. **(O)** Electron micrographs of cambial zone in wild type and p*COMT*:*PttACL5* lines C15 and C16 (left to right). Scale bar indicates 10 μm. For growth measurements, gene expression and CK, statistically significant difference is indicated with an asterisk when *p* < 0.05 (Student’s *t*-test).)

Growth of the transgenic lines carrying the p*COMT:PttACL5* construct was quite normal (Figure 5A). Height growth was slightly but significantly increased in three of the lines (Figure 5B), but no changes were observed in cambial growth compared to the wild type trees (Figure 5C). Similar to the POPACAULIS5 trees, it was not possible to obtain overexpression of *ACAULIS5*. The expression of *ACAULIS5* (derived from both the transgene and the endogenous gene) was not altered in the expanding xylem tissues in any of the lines (Figure 5D). In maturing xylem, however, the expression of *ACAULIS5* was suppressed with line C16 having the lowest expression (Figure 5E). The expression of the endogenous *PttACL5* gene was suppressed in all lines (Supplementary Figures S3E–G). The expression of *PttSAC51* correlated with the expression of *ACAULIS5* in most of the lines (Figures 5F,G). No consistent alterations were observed in the levels of cytokinins in two transgenic lines C15 and C16 (Figures 5H–M). Only the levels of *trans*-zeatin and its riboside precursor were increased in line C16, compared to the wild type (Figures 5H,I). Also wood anatomy was indistinguishable from the wild type, and no biseriate rays were observed in these trees (Figures 5N,O). In conclusion, reduction in the expression of *ACAULIS5* specifically in the xylem tissues did not consistently influence accumulation of cytokinins in these tissues or the activity of the cambium. The transgenic line C16 was most suppressed for the expression of *ACAULIS5* and had also increased levels of the *trans*-zeatin type cytokinins but these changes did not coincide with changes in the cambial growth of the stem. In conclusion, ACAULIS5 function in the maturing xylem is not expected to influence the activity of the vascular cambium, as suppressed expression of *ACAULIS5* in these tissues did not influence cambial growth.

## Discussion

Low level of *ACAULIS5* expression inhibited cytokinin biosynthesis and cambial growth of the transgenic trees carrying the *35S:POPACAULIS5* construct (Figures 1, 4). The effect was presumably derived from ACAULIS5 function in phloem or cambial tissues as transgenic trees where *ACAULIS5* expression was specifically modified in secondary xylem tissues did not show a decrease in cambial growth (Figure 5). From this follows that ACAULIS5 is required for normal cambial activity of the stem. This is opposite to the role of ACAULIS5 in suppressing cell division activity in the vascular tissues of the primary root, leaves and inflorescence stem of *Arabidopsis* (Hanzawa et al., 1997; Clay and Nelson, 2005; Baima et al., 2014; Vera-Sirera et al., 2015). On the other hand, ACAULIS5 is required to sustain secondary growth from the vascular cambium in the *Arabidopsis* hypocotyl. We observed earlier that the *Arabidopsis acl5* mutant does not form continuous vascular cambium and is halted in secondary growth after the initial stimulation of cell division activity of the root and the hypocotyl (Muñiz et al., 2008). Low cambial activity in trees with low *ACAULIS5* expression coincided in the current study with low cytokinin levels, suggesting positive effect of ACAULIS5 on cytokinin biosynthesis (Figure 4). This effect is specific to the vascular tissues as increased levels of *ACAULIS5* in leaf tissues did not result in increased cytokinin levels (Figures 3, 4). Notably, the stimulatory effect of ACAULIS5 on cytokinin levels of the stem vascular tissues is in sharp contrast to findings in the vascular tissues of the primary root and young hypocotyls of *Arabidopsis* where ACAULIS5 suppresses cytokinin biosynthesis by stimulating translation of SAC51, thereby inhibiting TMO5/LHW action on the cytokinin biosynthetic *LOG* genes. It seems therefore that ACAULIS5 function differs during different stages of vascular development. A phase change is known to occur in vascular development of *Arabidopsis* hypocotyl at the time of bolting when vascular expansion is highly stimulated and xylem fibers are formed (Sibout et al., 2008; Milhinhos et al., 2019). Both these processes, xylem expansion and fiber formation, are missing in the hypocotyl of the *acl5* mutant (Muñiz et al., 2008). It seems therefore that ACL5 stimulates cytokinin accumulation and cambial growth specifically at phases of strong xylem expansion, such as those experienced in *Arabidopsis* hypocotyls after bolting or during secondary growth of tree stems.

In addition to changes in secondary growth, the morphology of the rays was altered in the stem tissues of the POPACAULIS5 trees (Figure 2). Instead of the strictly uniseriate rays that are characteristic to the whole *Populus* genus, biseriate rays were occasionally initiated in the cambial zone. This phenotype could be due to increased anticlinal cell division activity in the ray initials. Alternatively, these trees display excessive anticlinal divisions of the fusiform initials. New fusiform initials that are formed as a result of anticlinal divisions can be transformed into ray initials (Larson, 1994), and it is possible that this process is affected in the POPACAULIS5 trees. Increased abundance of anticlinal divisions is surprising considering the low cytokinin concentration of the cambial region of these trees. It raises an interesting question on whether cytokinins could inhibit anticlinal cell divisions while promoting the periclinal divisions of the cambial cells. Presence of biseriate or multiseriate rays have been reported in *Populus* in response to conditions stimulating ethylene production (Savidge, 1988), and it is possible that deviation from the uniseriate ray structure involves interactions with other hormonal pathways.

Interestingly, it was not possible to overexpress *ACAULIS5* or increase thermospermine levels in the stem tissues of hybrid aspen. Similar situation was observed earlier in *Arabidopsis* stems where the expression of the *ACL5* transgene was counteracted by suppression of the expression of the endogenous *ACL5* (Baima et al., 2014). We suggested earlier a model according to which *ACAULIS5* expression is tightly balanced through a negative feedback regulation including the homeobox transcription factor HOMEOBOX PROTEIN8 (HB8) and auxin (Milhinhos et al., 2013). Also, the work of Baima et al. (2014) was supportive of such feedback regulation. In addition, they showed that *Arabidopsis* HB8 (AtHB8) directly controls the expression of *ACL5* in *Arabidopsis*. AtHB8 together with the four other HD Zip III transcription factors of the same clade, were recently shown to define xylem identity and direct cell divisions in the adjacent cambial cells downstream of auxin signaling (Smetana et al., 2019). It is therefore possible that the low auxin levels and low *HB8* expression in the POPACAULIS5 trees, demonstrated in our previous study (Milhinhos et al., 2013), are causally related to the low cell division activity of these trees. As both cytokinin and auxin levels were altered in the POPACAULIS5 trees, it is not possible with the current knowledge to distinguish which of these alterations underlies the changes in cambial growth. However, the collective evidence from *Arabidopsis* supports a direct effect of ACAULIS5 on both the auxin and cytokinin signaling (Baima et al., 2014; Vera-Sirera et al., 2015). We therefore propose that ACAULIS5 functions to integrate the two signaling pathways during secondary growth, guaranteeing appropriate cell division activity as well as xylem differentiation.

## Data Availability Statement

The original contributions presented in the study are included in the article/Supplementary Material, further inquiries can be directed to the corresponding author.

## Author Contributions

AM and HT conceived the study, designed the work with input from all authors, and prepared the manuscript draft. AM, BB, ON, and HT performed the experiments and analyzed the data. MB, ON, and CM critically reviewed the manuscript. All authors read and approved the final manuscript.

## Conflict of Interest

The authors declare that the research was conducted in the absence of any commercial or financial relationships that could be construed as a potential conflict of interest.

## Supplementary Material

Supplementary Figure 1 SAC51 homologs in *Populus*.

Supplementary Figure 2 Biseriate ray cells phenotype.

Supplementary Figure 3 Relative expression of *ACAULIS5* in *35S*:*POPACAULIS5* and p*COMT:PttACL5* transgenic lines tissues.

Supplementary Figure 4 GUS activity in TCSn:GUS transgenic lines in the POPACAULIS5 tree background.

Supplementary Table 1 Complete list of cytokinin levels in wild type and *35S:POPACAULIS5* and p*COMT:PttACL5* transgenic lines.

Supplementary Table 2 Oligonucleotides used in this study.
