# Supplementary material for: ACAULIS5 Is Required for Cytokinin Accumulation and Function During Secondary Growth of Populus Trees
Source: Front Plant Sci. 2020 Nov 16;11:601858. doi: 10.3389/fpls.2020.601858 (PMC7701098; doi:10.3389/fpls.2020.601858)
Supplement: Supplementary Figure 1 — SAC51 homologs in Populus. [file Data_Sheet_1.pdf]

Supplementary Figures. Milhinhos *et al.* ACAULIS5 is required for cytokinin accumulation and function during secondary growth of *Populus* trees

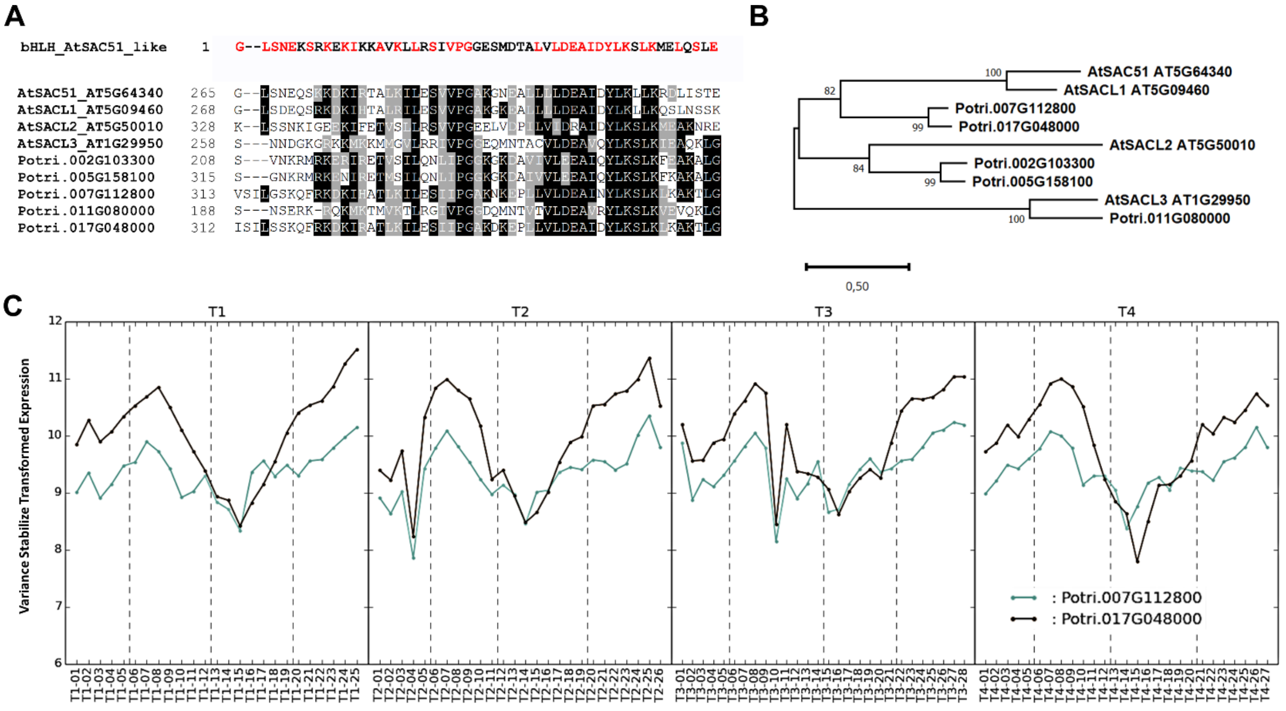

Supplementary Figure S1. *Populus* SAC51 gene family. (A) Alignment detail of amino acid sequences at the conserved SAC51_like specific basic helix-loop-helix (bHLH) domain site (bHLH_AtSAC51_Like domain indicated in red, cd:18917; Lu *et al*., 2020) as found in *Arabidopsis thaliana* SUPPRESSOR OF ACAULIS 51 (SAC51), three SAC51 like (SACL1, SACL2, and SACL3) and *Populus trichocarpa* SAC51 and SAC51 like proteins. Gene ID and accession numbers are indicated for *Arabidopsis* and *Populus*. BLAST searches in Phytozome *Populus trichocarpa* JGI v3 (https://phytozome.jgi.doe.gov/pz/portal.html) and PopGenIE_v3 (http://popgenie.org/) for *Arabidopsis* SACL (SAC51 and SACL) genes identified five putative homologs for SACL in *Populus trichocarpa*, that are indicated (gene models Potri.007G112800, Potri.017G048000, Potri.002G10330, Potri.005G158100 and Potri.011G080000). Deduced amino acid sequences for SACL were used for the alignment. Dashes indicate gaps and numbers depict bHLH conserved domain start point. (B) Phylogenetic relationship of SACL proteins, shown in (A). The tree was inferred by using the Maximum Likelihood method and JTT matrix-based model using MEGAX software (Kumar *et al*., 2018). The tree with the highest log likelihood (-6060,20)

is shown. The tree is drawn to scale, with branch lengths measured in the number of substitutions per site. (C) Gene expression profiles for the homologs of SAC51 across developing phloem and wood-forming tissues from four aspen trees (T1-T4, *P. tremula*) in the AspWood database (http://aspwood.popgenie.org/aspwood-v3.0/, Sundell *et al*., 2017). The expression of both genes is high in the cambial zone (the first peak) as well as later during xylem differentiation (the second peak).

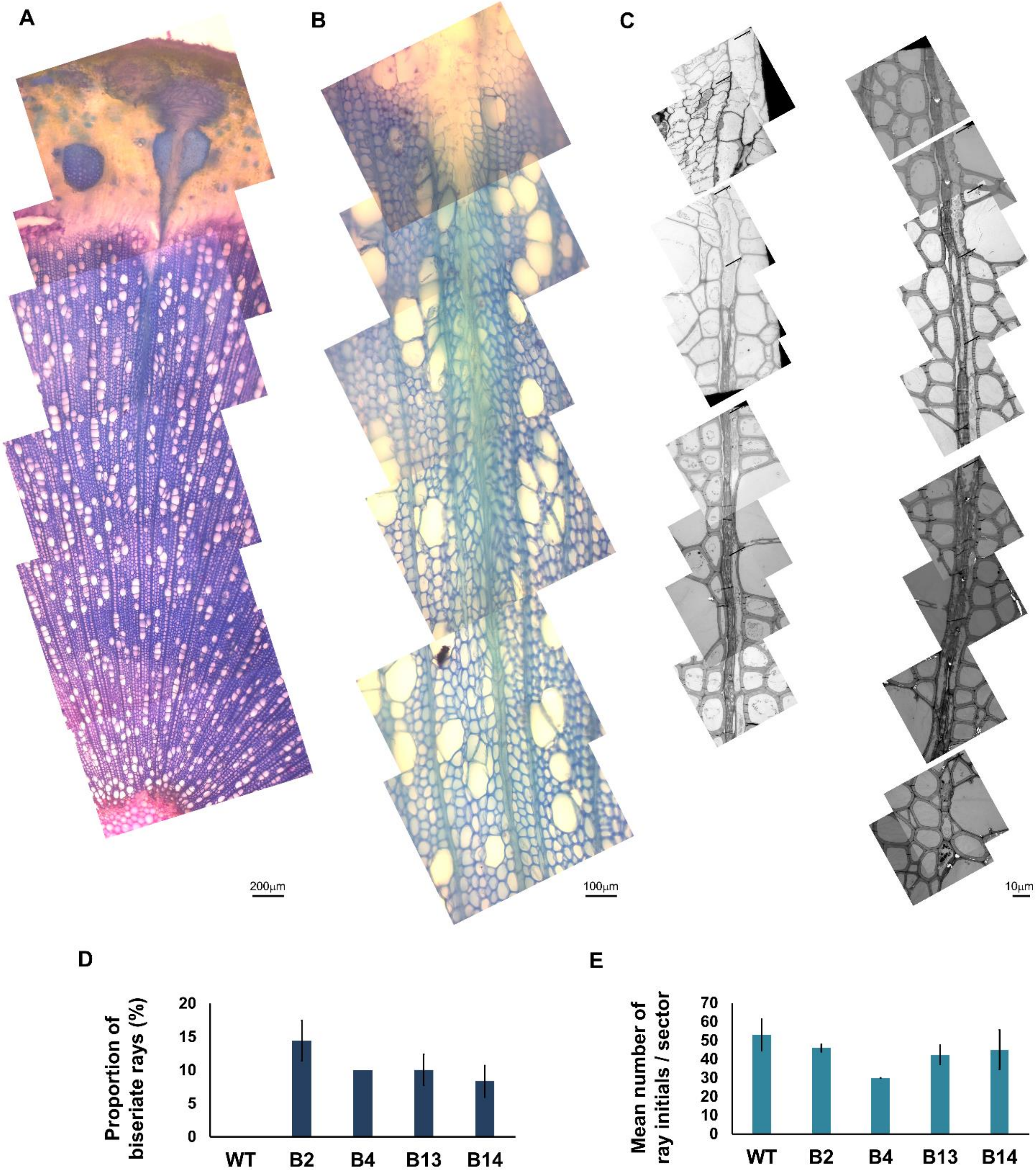

Supplementary Figure S2. Xylem anatomy in *Populus* stem sections of transgenic POPACAULIS5. (A) Light micrograph of a transverse section from the transgenic line B13 stem where biseriate rays are observed. (B) Detail of the cambial region and secondary xylem where biseriate rays are observed in stem sections of transgenic line B13. (C) Electron micrographs

tracing the biseriate ray phenotype across the stem in the transgenic line B13. (D) Percentage of biseriate rays in stem cross-sections of transgenic POPACAULIS5 lines. (E) Mean number of ray initials in quadrant sectors of cross sections of transgenic POPACAULIS5 and wild-type (WT) stems. Error bars depict ± S.D. Scale bars: 200 μm (A), 100 μm (B), 10 μm (C).

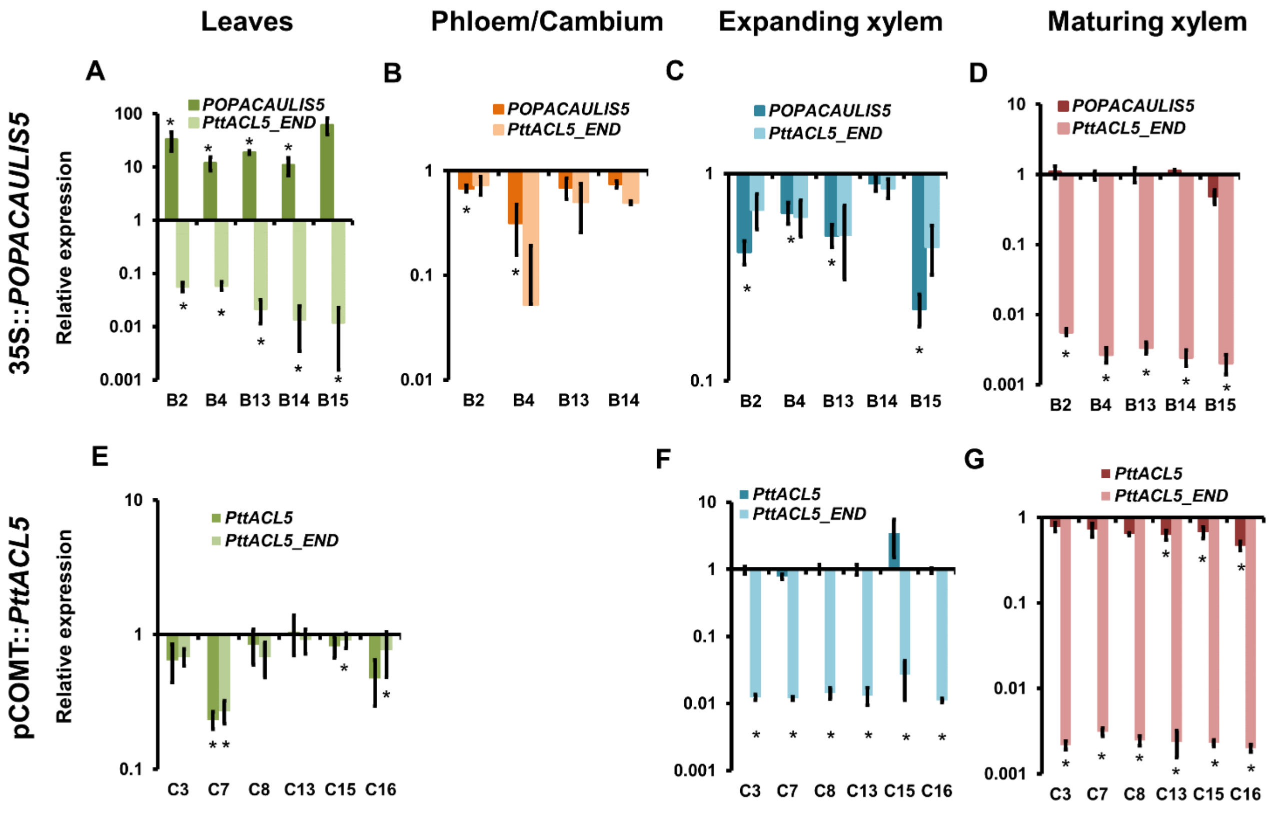

Supplementary Figure S3. Expression of ACAULIS5 in transgenic 35S::*POPACAULIS5* and pCOMT::*PttACL5* trees. The expression of the transgenes (*POPACAULIS5* and *PttACL5*) and the endogenous *PttACL5* (*PttACL5_END*) is shown in leaves (A, E), phloem/cambium (B), expanding xylem (C, F) and maturing xylem (D, G) of 35S::*POPACAULIS5* transgenic trees from lines B2, B4, B13, B14 and B15 (A-D) and of pCOMT::*PttACL5* transgenic trees from lines C3, C7, C8, C13, C15 and C16 (E-G). Observe that the *POPACAULIS5* (A-D) and *PttACL5* (E-G) transgene expression combines the expression from both the transgene and the endogenous *PttACL5* gene. Transcript levels are given relative to the wild-type level in each tissue. Error bars depict $\pm$ S.D. Statistically significant difference is indicated with an asterisk when $p<0.05$ (Student's t-test).

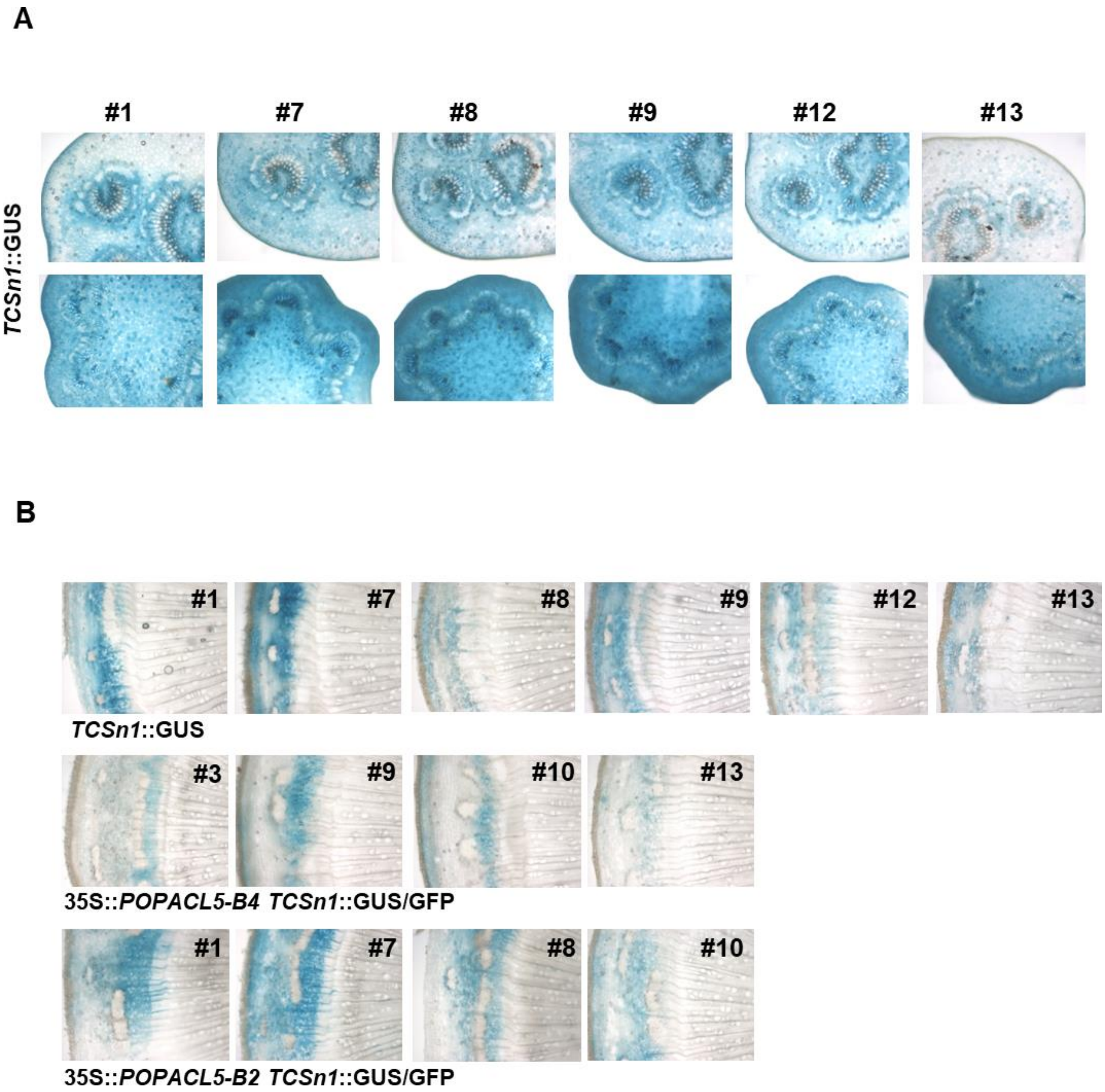

Supplementary Figure S4. Activity of a cytokinin reporter in POPACAULIS5 transgenic trees. The effect of POPACAULIS5 on the spatial distribution of cytokinin signalling was analysed in wild type and POPACAULIS5 lines B4 and B2 carrying a construct where the TCSn (Two component signalling sensor) was coupled to the GUS reporter gene. The activity of the TCSn was observed in stem tissues by histochemical GUS analysis in several transgenic lines. (A) TCSn::GUS activity in young tissues of the stem in the wild-type T89 background. (B) TCSn::GUS activity in wild-type (upper panel) and in POPACAULIS5 transgenic lines B4 and B2 (lower panels). # refers to the different double transgenic lines analysed.

References

Lu S, Wang J, Chitsaz F, Derbyshire MK, Geer RC, Gonzales NR, Gwadz M, Hurwitz DI, Marchler GH, Song JS, Thanki N, Yamashita RA, Yang M, Zhang D, Zheng C, Lanczycki CJ, Marchler-Bauer A. 2020. CDD/SPARCLE: the conserved domain database in 2020. Nucleic Acids Research **48,** D265-D268.

Kumar S, Stecher G, Li M, Knyaz C, and Tamura K. 2018. MEGA X: Molecular Evolutionary Genetics Analysis across computing platforms. Molecular Biology and Evolution **35,** 1547-1549.

Sundell D, Street NR, Kumar M, Mellerowicz EJ, Kucukoglu M, Johnsson C, Kumar V, Mannapperuma C, Delhomme N, Nilsson O et al. 2017. AspWood: high-spatial-resolution transcriptome profiles reveal uncharacterized modularity of wood formation in *Populus tremula*. The Plant Cell **29,** 1585-1604.
